# Aortic aneurysm with complete atrioventricular block and acute coronary syndrome

**DOI:** 10.1186/s13104-016-2050-2

**Published:** 2016-05-04

**Authors:** Moacyr Magno Palmeira, Hellen Yuki Umemura Ribeiro, Yan Garcia Lira, Fernando Octávio Machado Jucá Neto, Ivone Aline da Silva Rodrigues, Maitê Silva Martins Gadelha, Yuri Santana do Carmo

**Affiliations:** Division of Cardiology, Section of Urgency and Emergency, Department of Intensive Care, Fundação Pública Estadual Hospital de Clínicas Gaspar Vianna, 2000 Alferes Costa street, Pedreira, Belém, Pará 66087-660 Brazil; Division of Cardiology, Section of Urgency and Emergency, Department of Clinical Care, Universidade do Estado do Pará, 2623 Perebebuí street, Marco, Belém, Pará 66087-670 Brazil; 60 Amazonas square, Jurunas, Belém, Pará 66025-070 Brazil

**Keywords:** Cardiovascular diseases, Aortic aneurysm, Acute coronary syndrome, Atrioventricular block

## Abstract

**Background:**

Acute aortic dissection (AAD) is a highly lethal and prevalent cardiovascular emergency. AAD can develop into atrioventricular conductivity disorders caused by coronary artery dissection, with acute myocardial infarction (AMI) being the most frequent clinical sign. In many deceased patients, the diagnosis is not confirmed until autopsy, and 85 % receive the wrong therapy as a result of misdiagnosis.

**Case presentation:**

A 49-year-old male patient presenting with prolonged, intense and sharp precordial pain radiating to his back, as well as cold sweats, nausea and vomiting, was admitted to the cardiac emergency service. Thorax examination revealed normal bilateral breath sounds and a respiratory frequency of 24 incursions/min (SpO_2_ 97 %). Cardiac auscultation revealed a heartbeat that was rhythmic, regular, and bradycardic. There was a visible high-intensity pulsation in the suprasternal notch, a diastolic murmur audible at the aortic focus, and a fourth heart sound on auscultation. The patient was diagnosed with Stanford type A AAD, concomitant complete atrioventricular block, and impairment of the right coronary artery, progressing to acute coronary syndrome (ACS) and spontaneous rupture of the aortic aneurysm. After a hemodynamic study, the patient was transferred for urgent surgical treatment and passed away during the procedure.

**Conclusion:**

Physical examination is essential to be able to disregard AAD as the main cause of AMI. The consequences of a misdiagnosis can be fatal if thrombolytic or anticoagulant therapy is chosen as the initial treatment; therefore, surgery is the best treatment for aortic dissection.

## Background

Acute aortic dissection (AAD) is a highly lethal cardiovascular emergency with an incidence of 2000 new cases per year in the United States and 3000 in Europe. The rate of incidence is 5–30 per million people per year and the mortality rate is 3.2/100.000 per year [1].

Atrioventricular conductivity disorders caused by coronary artery dissection involving AAD are rare complications [2, 3]. Both coronary arteries can be affected, and acute myocardial infarction (AMI) is the most frequent clinical symptom [2].

We report a case of Stanford type A AAD with right coronary artery dissection, which developed into complete atrioventricular block and acute coronary syndrome (ACS).

## Case presentation

A 49-year-old male patient, a farmer, married, and living in Irituia City, presented at the cardiac emergency service with prolonged, intense, and sharp precordial pain radiating to his back. He also had intense cold sweats, nausea, and vomiting. The symptoms started 6 h prior to hospital admission. The patient was obese, hypertensive, and a long-time smoker. He was taking 25 mg of captopril daily. Diabetes, alcoholism, previous AMI, cerebrovascular accident, or any other comorbidity were denied. He was admitted in good general condition, hydrated, pale (++/4+), acyanotic, afebrile, mildly dyspneic, bradycardic, conscious, oriented in space and time, verbalizing well, and walking with assistance.

A thorax examination revealed normal bilateral breath sounds without adventitious sounds and a respiratory frequency of 24 incursions/min (SpO_2_ 97 %). Cardiac auscultation yielded a rhythmic, regular, and bradycardic heart rate (44 beats/min). There was a visible high-intensity pulsation in the suprasternal notch, a diastolic murmur audible at the aortic focus, and a fourth heart sound on auscultation. He had asymmetry of pulses in the upper limbs, with the right radial artery pulse stronger than the left. Blood pressure was 120/80 mmHg. His abdomen was distended and painful during examination. Bowel sounds were present on auscultation.

An electrocardiogram (ECG) revealed complete atrioventricular block and ST-segment elevation in the inferior wall. Therefore, the patient was treated for AMI using supportive measures, such as oxygen and initial thrombolytic therapy with tenecteplase 45 mg in a single bolus, to achieve reperfusion. After stabilization, the patient was sent for hemodynamic evaluation for urgent catheterization.

**Fig. 1:**
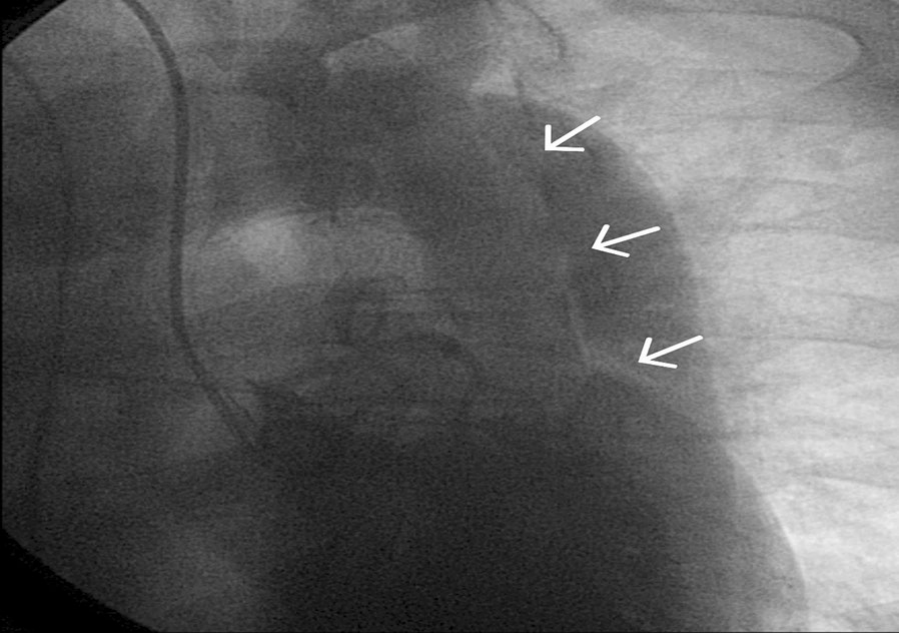
Aortic dissecting aneurysm. Coronary angiography revealed an aortic dissecting aneurysm during hemodynamic evaluation for urgent catheterization

**Fig. 2:**
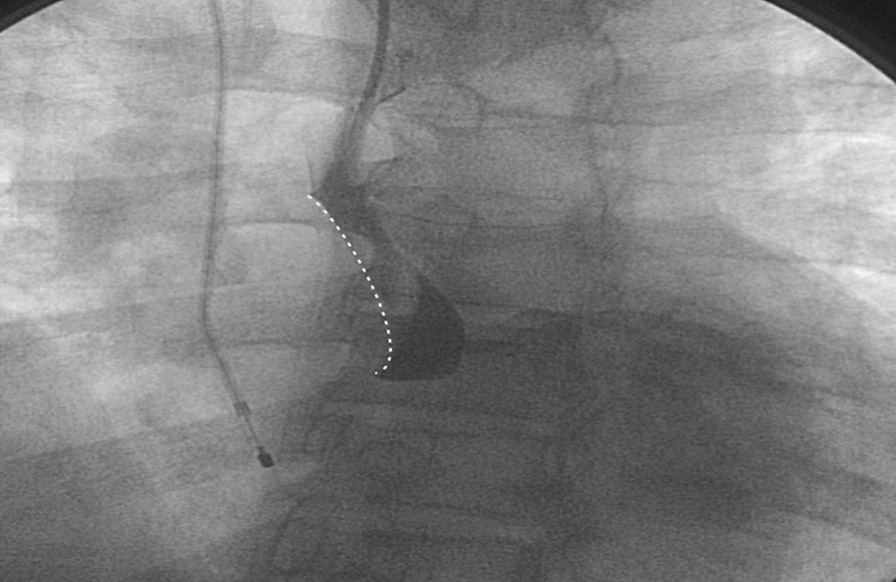
Right false coronary lumen area. Coronary angiography revealed a right coronary false lumen area caused by aortic dissecting aneurysm extending to the right coronary ostium

Coronary angiography showed an aortic dissecting aneurysm (Fig. 1) and a right coronary false lumen area (Fig. 2). The patient was diagnosed with Stanford type A AAD, concomitant complete atrioventricular block, and impairment of the right coronary artery, progressing to ACS and spontaneous rupture of the aortic aneurysm.

After a hemodynamic study, the patient was transferred for emergency surgical treatment. The surgery was performed through a median sternotomy. The initial inspection revealed aortic dilatation and visible aortic dissection in the aortic crest as well as in the proximal portion. There was an aneurysm rupture in the aortic right proximal portion with remnants clots. During these initial steps, the patient’s blood pressure decreased and failed to stabilize. Further, several bleeding foci presented and aortic cross-clamping was difficult due to dissection damage. The patient died during the procedure.

## Discussion

AAD is an acute lesion of the aortic wall, accompanied by separation of the intimal and medial layers of this artery, due to rupture or intramural hematoma [1]. It is a very serious vascular event with a high mortality rate. Further, many deceased patients remain misdiagnosed until autopsy. Therefore, a rapid diagnosis is vital to providing the appropriate treatment in a timely manner [2]. The dissection begins transversely, as it is located in the ascending aorta in 70 % of cases, with 20 % in the descending aorta after the aortic arch and 10 % in the transverse aortic arch. According to the Stanford classification, aneurysms in the ascending aorta are type A and aneurysms not involving the ascending aorta are type B [3]. Once started, further dissection can be distal, retrograde, or can occur in both directions, advancing in variable lengths [4].

The main symptom of AAD is severe chest pain, usually in the anterior region. The pain commonly has an abrupt onset and is of high intensity, usually maximum from the beginning of the dissection [5]. Similarly to AMI, acute dissection is usually associated with symptoms of profuse sweating, pallor, tachycardia, tremors, nausea, and vomiting [5].

ECG in AAD is often nonspecific, with approximately a third of them revealing changes that are consistent with left ventricular hypertrophy and another third appearing normal. Nevertheless, obtaining an ECG is important for three reasons. First, in patients with AAD presenting with unspecified chest pain, the absence of T waves and ST-segment ischemia may suggest another main diagnosis, aside from myocardial ischemia and including AAD, in patients with proximal dissection. Second, the ECG may reveal AMI when the dissection flap involves a coronary artery [6]. Third, it can reveal the coexistence of a cardiac rhythm disorder [7].

In AAD, rare and irregular rhythms may be present, such as bundle branch block and atrioventricular block. In Stanford type A dissections, it is suggested that the pathophysiological mechanism, which generates the rhythm disorder, is the presence of hematoma in the interatrial septum and/or in the atrioventricular junction, which results in cardiac block [7].

In our case, the ECG revealed complete atrioventricular block and changes suggestive of AMI with inferior wall involvement. Coronary artery dissection in the course of AAD is usually diagnosed by the presence of AMI or by identifying the displacement of the intimal layer through coronary angiography [5]. In a small minority, approximately 1–2 % of the cases [8], a proximal dissection flap may involve the ostium of a coronary artery and cause myocardial infarction, which can hinder the diagnosis. The dissection more often affects the right coronary artery than the left, explaining why these myocardial infarctions tend to have a lower location [6]. Unfortunately, when there is a secondary AMI, the symptoms may lead to a misunderstanding of the primary symptoms of AAD and complicate the clinical picture. The possibility that within the electrocardiographic evidence of myocardial infarction, the existing AAD may remain unrecognized is even more worrisome. Therefore, physical examination is essential to be able to disregard AAD as the main cause of AMI. Asymmetry of the peripheral pulses is found in 50 % of type A dissections. A diastolic murmur audible at the aortic focus with normal valves contributes to an AAD diagnosis, and the presence of abdominal sounds, despite the abdomen being distended and painful, could suggest that there is no involvement of the descending aorta or global damage[3–5].

The diagnosis of AMI and AAD can occur concomitantly in 1.5–7.5 % of cases. As surgery is the best treatment for AAD, the consequences of a misdiagnosis within this syndrome can be fatal if thrombolytic therapy is chosen as the primary treatment. Moreover, 85 % of the patients receive the wrong therapy as a result of misdiagnosis [2, 4–6]. In a literature review, among 21 cases of AAD that were treated with thrombolytic therapy, early mortality was reported in 71 % cases, of which many deaths were caused by cardiac tamponade [9].

The very high risk of cardiac tamponade and other complications has led to a consensus that surgery is superior to clinical management for acute type A dissections. While surgical repair of the ascending aorta greatly reduces such risk, some of the patients referred to surgery are already in a very serious condition, which reduces their chances of survival, not because of surgical complications but as a result of their serious medical condition [2].

## Conclusion

As the symptoms of a secondary AMI can lead to a misunderstanding of the primary symptoms of AAD, physical examination is essential in being able to disregard AAD as the main cause of AMI. The consequences of a misdiagnosis can be fatal if thrombolytic or anticoagulant therapy is chosen as the initial treatment. Therefore, surgery is the best treatment for aortic dissection.

## Abbreviations

AAD: acute aortic dissection
ACS: acute coronary syndrome
AMI: acute myocardial infarction
ECG: electrocardiogram
